# Effectiveness of Two Intensity Levels of Diode Laser in Debonding Metallic Brackets Regarding Enamel Surface Integrity and Pulpal Temperature: An Ex-Vivo Study

**DOI:** 10.7759/cureus.41372

**Published:** 2023-07-04

**Authors:** Mudar Mohammad Mousa, Hiba M.G. Al-Jannan, Kinda Sultan, Mowaffak A Ajaj, Mohammad Y Hajeer, Ahmad Al-Manadili, Ali Mohsen Ammar, Mohammed Awawdeh

**Affiliations:** 1 Department of Orthodontics, Faculty of Dentistry, University of Damascus, Damascus, SYR; 2 Department of Oral Histopathology, Damascus University, Damascus, SYR; 3 Department of Orthodontics, Faculty of Dentistry, Arab Private University for Science and Technology, Hama, SYR; 4 Department of Preventive Dental Science, College of Dentistry, King Saud Bin Abdulaziz University for Health Sciences, Riyadh, SAU

**Keywords:** laboratory research, pulpal temperature, enamel cracks, debonding, diode laser, metal bracket

## Abstract

Introduction: The traditional methods of deboning metal brackets exert excessive force, resulting in enamel scratches, fractures, and patient discomfort. The objective of this study was to evaluate the effectiveness of using two intensity levels of a diode laser for debonding metallic orthodontic brackets as an alternative to the conventional debonding method.

Materials and methods: Sixty intact, extracted human premolar teeth were used in this study, and metal orthodontic brackets were bonded to the buccal surface of these teeth. The teeth were divided into three groups for the experiment: (1) the control group, where conventional bracket debonding was performed using a debonding plier, (2) the first experimental group, where a diode laser (2.5W, 980nm) was utilized for laser debonding, and (3) the second experimental group, where a diode laser (5W, 980nm) was used for laser debonding. The laser was applied using a sweeping movement for 5 seconds. After debonding, the adhesive remnant index (ARI), the lengths, and the frequency of enamel cracks were compared among the groups. Additionally, an increase in intra-pulpal temperature was measured.

Results: In all groups, there were no instances of enamel fractures. Laser debonding resulted in a significant reduction in both the frequency and length of newly formed enamel cracks compared to the conventional debonding method. The laser debonding group exhibited increases in intra-pulpal temperature of 2.37°C and 3.60°C in the second and third groups, respectively. These temperature increases were significantly lower than the threshold of 5.5°C. There were no significant differences observed in the ARI scores among the groups.

Conclusion: With all debonding methods, an increase in the length and frequency of enamel cracks should be anticipated. However, laser-assisted debonding of metal brackets offers the advantage of reducing the risk of enamel damage while avoiding thermal damage to the pulp.

## Introduction

Although the bonding materials must provide enough bond strength to resist intraoral and orthodontic forces, the bond strength should not be that high during bracket debonding to avoid enamel fracture [1]. During the process of removing brackets, bond failure can occur at either the adhesive-enamel interface or the adhesive-bracket interface, resulting in adhesive failure. Bond failure can also occur within the adhesive itself, leading to cohesive failure [2]. The combination of adhesive and cohesive failure (mixed failure) is generally the most common type [3]. Usually, the adhesive failure between resin and enamel surface is considered to be the riskiest and most damaging to surfaces of teeth, which occurs especially when using ceramic brackets, but enamel fracture could also happen with metal brackets [4]. The effects caused by bracket removals like ruptures, cracks, avulsion fractures, or any small-scale enamel damage are important factors in the caries development process [5,6].

To ensure that the enamel surfaces return to their original state before orthodontic treatment, it is necessary to remove composite remnants from the tooth surfaces after treatment termination [7]. The literature describes various techniques for debonding metal and ceramic brackets, including special debonding pliers, ultrasound, or laser applications [1]. There are four major types of lasers in dentistry. Laser types are primarily classified based on their lasing mediums, which are categorized by their state: gas, liquid, solid, and semiconductor (also known as laser diode) [8]. A diode laser, also called an injection laser or a laser diode, is a semiconductor device that generates coherent radiation within the visible or infrared spectrum when an electric current passes through it [8].

In a review of the existing literature, Ghazanfari et al. have mentioned that irradiation from various types of lasers, including Nd:YAG, Er:YAG, CO2, Tm:YAP, diode, or ytterbium fiber lasers, can effectively reduce the shear bond strength of ceramic brackets and accelerate the debonding process. According to the authors, this method offers a secure method for removing ceramic brackets with little negative impact on the intrapulpal temperature and enamel surface, decreasing the incidence of ceramic bracket failure [9]. Additionally, Feldon et al. found that diode lasers can reduce the force required for debonding monocrystalline ceramic brackets without significantly raising pulp temperature [8]. Using an Er:YAG laser, Oztoprak et al. showed that the force required to remove polycrystalline ceramic brackets could be reduced [10].

In comparison with metallic brackets, there is a greater risk of fracturing the enamel surface during ceramic bracket debonding, but as shown in recent scanning electron microscopy investigations, there is a risk of enamel damage during debonding of metallic brackets as well [11,12]. However, the use of lasers was not limited to ceramic bracket debonding but rather to metallic bracket debonding. Sedky et al. found that Er, Cr: YSGG laser irradiation was effective in debonding stainless steel orthodontic brackets and reducing adhesive remnants after the debonding process [13]. Lesniak et al. suggest that Er: Yag laser irradiation revealed no damage to enamel after laser debonding of both metallic and ceramic brackets compared to mechanical methods [14].

According to Knaup et al., using a 445-nm diode laser for irradiating metallic brackets before debonding does not significantly reduce shear bond strength (SBS) values. Additionally, it does not impact the amount of adhesive that remains on the enamel surface. They said it was unclear whether different wavelengths in the laser or bracket systems might lead to different results, and further research was needed [15]. Therefore, other wavelengths of diode lasers have not been investigated yet, such as the 980-nm diode laser in debonding metal brackets. Therefore, the current study aimed to investigate the effect of diode laser application on debonding stainless steel brackets. The research hypothesis was that diode laser irradiation affected the debonding of metallic orthodontic brackets.

## Materials and methods

Study design and settings

This comparative interventional-controlled in vitro study was performed at the Laser Research Unit, Faculty of Dentistry, Damascus University. The Local Research Ethics Committee of the Faculty of Dentistry, University of Damascus, reviewed and approved the protocol of this study (Approval No. UDDS-228-2019HG/SRC1952), which was funded by the University of Damascus Postgraduate Research Budget (Ref No: 863082063DEN).

Sample size calculation

G*Power software, version 3.1.3 (Universität Düsseldorf, Düsseldorf, Germany), was used for sample size calculation. The effect size for pulpal thermal change based on the results of Nalbantgil et al. was 0.571° C [16]. Using one-way ANOVA as a statistical test with a significant level of 0.05 and a power of 0.95, the calculation showed the need for 60 extracted premolars. The maximum mean difference for enamel crack length based on the results of Heravi et al. was 3.5 mm [17]. Employing one-way ANOVA as a statistical test with a significant level of 0.05 and a power of 0.95, the calculation revealed the need for 60 extracted premolars.

Sample collection

The principal researcher (H.A.) investigated patients enrolled at the Department of Orthodontics at the Faculty of Dentistry of Damascus University between January 2020 and December 2021. Forty-five patients who met the inclusion criteria underwent clinical and radiographic examinations, and all signed a written consent form after being informed about the use of their extracted teeth. The patients' inclusion criteria were as follows: (1) residents of Damascus, (2) aged 18 to 25, (3) had no prior orthodontic treatment, and (4) needed upper premolar extraction as part of their orthodontic treatment plan.

Ninety upper premolars were extracted during the study period, of which 73 met the inclusion criteria. Of these, 60 premolars were chosen randomly and included in the study. The premolars' inclusion criteria were: (1) freshly extracted premolars for orthodontic reasons, (2) intact buccal surfaces with no enamel defects, such as visible cracks, restorations, hypoplastic lesions, or fractures. The premolars' exclusion criteria were as follows: (1) premolars previously treated with orthodontic brackets, (2) premolars exposed to whitening or fluoridation treatment at least two weeks before extraction, and (3) the presence of more than one vertical crack.

Teeth preparation and the preliminary imaging

The extracted premolars were cleaned with tap water, a manual scaling tool was used to remove gingival remnants, ligamentous fibers, and calculus without the use of any chemicals, and they were then stored at room temperature in thymol solution 0.2% to inhibit any bacterial growth until their use. Using a spherical diamond bur (1016, KG Sorensen, São Paulo, Brazil). A 2-mm-diameter cavity was made from the occlusal surface following the pulp chamber. A manual curette was used to remove the pulp tissue debris, and 1% sodium hypochlorite was used as an irrigation solution. After that, the extracted premolars were polished using fluoride-free pumice (Nada™ Pumice Paste; Preventech, Matthews, NC, USA) and examined under a 20-X magnification stereoscope (Model SKT 41322, Meiji Techno, Japan) to ensure the absence of caries and cracks on the labial surface. Several pictures of each tooth's labial surface were taken using a digital camera with a resolution of 30.4 megapixels (Canon 5D Mark IV, Ota City, Tokyo, Japan) under a 20-X magnification stereoscope, then transferred to a computer to determine the length and direction of cracks, if any, using the Autodesk Revit® 2017 program (Autodesk, Inc., San Francisco, California, USA).

Bonding procedures

The labial surface of each tooth was covered with Teflon tape so that only the area requiring etch preparation appeared to prevent any unwanted etching or harm. The area beneath the bracket base was estimated to be within 9.9 mm2. Then they were etched using 37% phosphoric acid gel for 30 seconds, rinsed with water for 15 seconds, and dried with air/oil-free compressed air until a chalky white appeared on the enamel surface. A thin layer of primer (Reselience®, OthoTechnology ™, Florida, USA) was applied to the etched enamel surface using a clean brush. A small amount of Reselience® adhesive paste (Otho technology ™, Florida, USA) was applied onto the bracket base (Votion®, MBT Compatible 0.022, Otho technology ™, Florida, USA), then adjusted to its exact position on the labial surface by pushing gently. Excess adhesive surrounding the bracket was removed with a sharp scaler without changing the position of the bracket. The adhesive was then cured for 40 seconds (10 seconds for each side) using a Woodpecker ® light curing unit (Guilin Woodpecker Medical Instrument Co., China), keeping the distance between the radiation source and the bracket constant (approximately 2 mm). The teeth were then stored in an incubator in a 0.2% thymol solution at 37°C. After seven days, the premolars were randomly divided into three groups (20 per group). One of the academic staff members who was not involved in this study created the randomization and sequence. Minitab® exported a list of randomly generated numbers with a 1:1:1: allocation ratio (version 17, Minitab, LLC, State College, PA) according to the presence or absence of the laser irradiation method during bracket removal into three groups: The first group (the control group) depended on the use of a bracket remover plier only (Chifa, KP-013-135-PMK, Nowy Tomysl, Poland), whereas the second and third groups (i.e., the experimental groups) included the use of 2.5-W and 5-W diode lasers, respectively.

Pilot study on the best parameters of laser irradiation and pulp temperature

Before initiating the laser application, a survey was conducted on 20 extracted first premolars, specifically for orthodontic purposes. The primary objective of the pilot study was to establish a safe and effective range of laser duration, frequencies, and power for diode lasers in debonding metal brackets; the temperature of the dental pulp was measured using a temperature sensor attached to a type K thermocouple (Zhangzhou Weihua Electronic Co., Fujian, China). This measurement was intended to demonstrate the extent of temperature alteration experienced by the dental pulp during laser application. This pilot study showed that the temperature in the pulp chamber increased by a range of 1.2°C to 4.3°C, with a mean of 2.4°C and a standard deviation of 0.88. Based on these results, the laser pattern was set to continuous, and the exposure time was fixed at 10 seconds. In addition, two different power capacities, 2.5 and 5 watts, were used to compare their effects on the temperature of the dental pulp and the formation of enamel cracks.

The first experimental group used a diode laser with a wavelength of 980 nm at a power of 2.5 W and a continuous mode applied in contact with the metal bracket with a sweeping motion for 10 seconds. Then the bracket was removed with the remover plier (Chifa™, KP-013-135-PMK, Nowy Tomysl, Poland). The instrument was placed between the tooth surface and the bracket base. Then it was squeezed gently to deform the wings of the bracket and remove the bracket. The second experimental group also used a diode laser with a 5 W output, whereas the third group was the control group without laser irradiation. In this group, the brackets were removed conventionally.

Outcome measures

Primary Outcomes: The Length, Direction, Number, and Formation of Cracks

**Figure 1:**
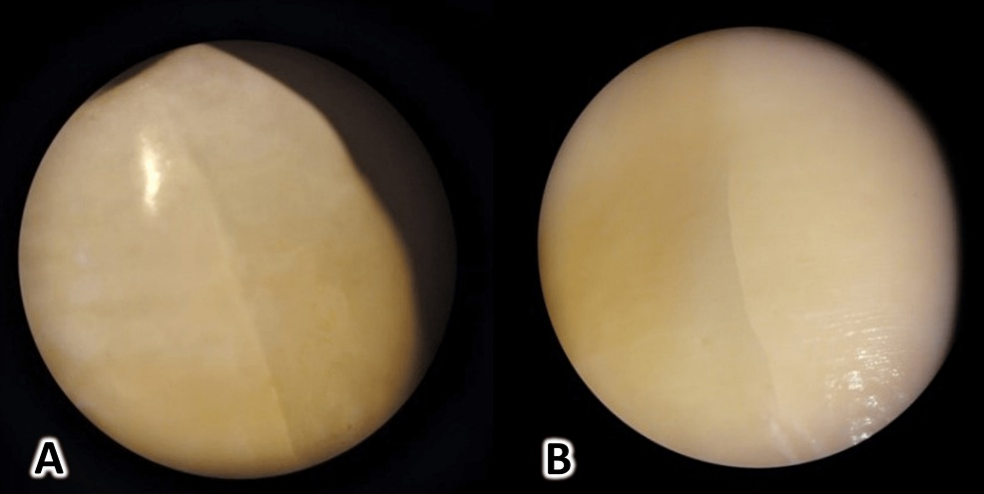
New crack formation after debonding metallic brackets: A and B show vertical cracks observed on the surface of the enamel.

After the removal of remnant adhesive from enamel surfaces using 12 tungsten carbide bur blades (Komet®, Gebr. Brasseler GmbH & Co., Germany), all teeth were photographed under the same conditions as the first photograph (Figure 1). The captured images were transferred to Autodesk Revit® software (Autodesk, Inc., San Francisco, California, USA). Crack length, number, and new crack formation were determined according to the following scale [18]: 1: enamel surface free from cracks or new cracks; 2: lengthening of pre-bonding cracks; 3: enamel surface with new cracks; 4: new cracks on the enamel surface and lengthening of pre-bonding cracks. The changes in the direction of enamel cracks were classified as follows [17]: Vertical (0-30 degrees to the long axis of the crown); Oblique (31-45 degrees to the long axis of the crown); Horizontal (46-90 degrees to the long axis of the crown); Mixed (when a crack had an altered direction).

**Figure 2:**
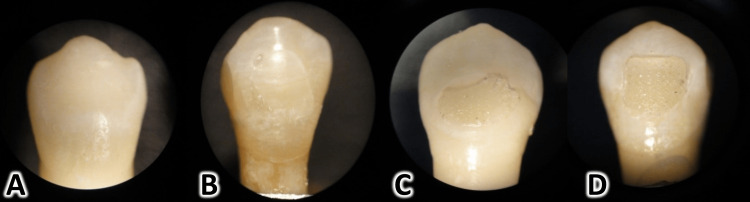
The method of scoring the adhesive remnant on the enamel surface (the Adhesive Remnant Index [ARI]). A: Score of 0 is given when all adhesive remnants are removed with the bracket. B: Score 1 is given when adhesive remnants cover less than 50% of the former bracket site. C: Score 2 is given when adhesive remnants cover over 50% of the former bracket site. D: Score 3 is given when all adhesive remnants are left on the tooth surface with a clear imprint of the bracket base.

The score of the Adhesive Remnant Index (ARI). The ARI was determined immediately after bracket removal using a stereomicroscope (SKT 41313, Meiji Techno, Saitama, Japan) at X-20 magnification [19]. After the visual inspection, the ARI scores ranged as follows: 0: all adhesive was removed with the bracket; 1: adhesive residuals covered less than 50% of the former bracket site; 2: adhesive residuals covered more than 50% of the former bracket site; and 3: adhesive residual was on the tooth surface with a clear imprint of the bracket base (Figure 2).

Secondary Outcomes: The Pulp Chamber Temperature

The change in pulp chamber temperature was measured using a type K thermocouple (Zhangzhou Weihua Electronic Co., Fujian, China), which was inserted into the pulp chamber and connected to a digital thermometer. The greater difference in the pulp chamber temperature was recorded during laser application.

Statistical analysis

IBM Corp. Released 2016. IBM SPSS Statistics for Windows, Version 24.0. Armonk, NY: IBM Corp. was used for statistical analysis. The Kolmogorov-Smirnov test was applied to check the normality of the data distribution. Statistical differences were studied by analyzing the variance (ANOVA) test to study the significant differences in the average length of cracks before and after bonding and the average length of newly formed cracks between the three studied debonding methods. Then, when the analysis of the ANOVA test showed significant differences, pairwise comparisons were carried out using the Tukey post-hoc test. The Kruskal-Wallis test was performed to detect the significant differences in the distribution of ARI and enamel damage index scores between the study groups. The Mann-Whitney U test was also performed to test for significant differences in the scores of each ARI and the enamel damage index. Moreover, the chi-square test was used to detect significant differences in the frequencies of the crack direction. On the other hand, a t-test was conducted to study the difference in temperature change during the removal of the brackets between the groups using the diode laser with 5 W and 2.5 W. The statistical significant level was set at an alpha level of 0.05.

## Results

No significant differences existed in the number and length of enamel cracks among the study groups before bracket bonding (P≈1 and P=0.694, respectively; Table 1). The traditional bracket removal group showed the greatest mean length of newly formed cracks, followed by the 2.5 W and 5 W diode groups (Mean length= 1.792, 1.431, 0.911, respectively). There was a significant statistical difference in the mean length of cracks formed after the brackets were removed between the study groups (P < 0.001).

**Table 1:** The number and length of enamel cracks.

Group	Lengths of enamel cracks		Number of enamel cracks		
	Before debonding	After debonding (new cracks)	Before debonding	After debonding (new cracks)	
	Mean ±SD	Mean ±SD			
Traditional debonding	2.109 ± 1.036	1.792 ± 0.406	18	14	
2.5W Diode	1.895 ± 1.556	1.431 ± 0.478	16	9	
5W Diode	2.109 ± 1.383	0.911 ± 0.375	17	7	
P-value	0.694	<0.001	≈1	0.867	

When performing pairwise comparisons, the mean length of cracks was significantly greater in the traditional bracket removal group than in the 5W diode group (P < 0.001). After brackets were removed, the traditional bracket removal group exhibited more new enamel cracks and greater frequencies of vertical (n=6) and mixed (n=6) cracks compared to the laser-aided debonding groups. However, these differences were not statistically significant between the three groups (P = 0.867, Table 2).

**Table 2:** The direction of newly formed enamel cracks in the three groups * Chi-square test.

	Vertical	Oblique	Horizontal	Mixed	Total	P-value*
Traditional debonding	6	2	0	6	14	0.867
2.5W Diode	3	0	3	3	9	
5W Diode	2	2	0	3	7	
Total	13	4	3	12	32	

When comparing the ARI score among the three groups, 75% of the sample in the traditional bracket removal group showed the greatest frequencies of the ARI score at (0) and (1). In the diode groups, greater frequencies were observed for scores (2) and (3). However, the differences between the three groups in the distributions of the ARI scores were not statistically significant (P = 0.226; Table 3). The increase in the inter-pulpal temperature was significantly greater in the 5-watt diode debonding group than in the 2.5-watt laser debonding group (mean temperature change = +3.60°C, +2.37°C, respectively; P = 0.15).

**Table 3:** Proportions of the ARI scores in the three groups following debonding. * Chi-square test

	ARI score				p-value*
Groups	0	1	2	3	
Traditional debonding	8 (40%)	7 (35%)	3 (15%)	2 (10%)	
2.5W Diode	3 (15%)	5 (25%)	6 (30%)	6 (30%)	0.226
5W Diode	3 (15%)	4 (20%)	5 (25%)	8 (40%)	

## Discussion

Orthodontic bracket debonding often presents clinical challenges due to the mechanical environment governing intraoral debonding [20]. When an orthodontic bracket is debonded, adhesive residuals may remain on the enamel surface, and there is a possibility of enamel cracking or enamel detachment during the debonding process [21]. Numerous methods for debonding have been proposed, such as ultrasonic debonding, electrothermal debonding [22], and using specially designed mechanical instruments for debonding [8,23]. Moreover, the experimental use of lasers with various wavelengths for ceramic bracket debonding has been documented since the 1990s. This study investigated the effect of laser-aided debonding of metal orthodontic brackets using a 980nm diode laser. The low cost and small size make diode lasers favorable for orthodontic practice [8].

The length, direction, and number of newly formed cracks following debonding

After brackets were debonded, the percentage of vertical cracks was 41%, mixed cracks were 37%, and horizontal cracks were 12% of the total crack orientations, regardless of the debonding method used. However, there were no statistically significant differences in newly formed cracks in different orientations after debonding the brackets between the three study groups. The conventional bracket removal group showed a greater rate of crack formation after debonding. This can be explained by the fact that the distribution of forces on the enamel surface may play a role in changing the crack direction or maintaining its original direction before debonding [4]. The decrease in crack formation rate in the laser groups can be attributed to the laser's thermal softening effect on the adhesive material, which weakens the bond strength between the surface, the enamel, and the bracket, resulting in lower stresses formed during debonding and consequently leading to less crack formation compared to the conventional method.

This result differed from a study by Heravi et al., which found that 90% of the cracks after debonding were mixed. The reason for this discrepancy may be that most of the cracks in the current study were vertical before bracket bonding, while in the study by Heravi et al., most of the cracks followed one or two directions, and only 25% of the cracks were vertical before bonding [17]. Statistical analysis revealed a significant increase in the average crack length in the conventional bracket group compared to both experimental groups. This difference was particularly noticeable between the 5-Watt diode and conventional bracket groups. However, there were no significant differences between the 2.5-Watt diode group, the 5-Watt diode group, and the conventional bracket group regarding the average crack length change before and after bracket debonding. Nevertheless, the increase in crack length was lower in the 5W diode group compared to the other study groups due to the laser's role in the thermal softening of the adhesive material, which increases with greater laser power.

The results of this current study differ from the findings of Habibi et al., who reported no increase in crack length after bracket debonding. This discrepancy may be attributed to the fact that bracket debonding in the Habibi et al. study was performed mechanically using a universal testing machine after 48 hours of bonding, as lab-based debonding forces are typically unidirectional, unlike manually applied debonding forces, which are often combinations of forces from various directions [24]. Furthermore, this study aligns with the findings of Heravi et al., who reported a statistically significant increase in crack length after bracket debonding. The average crack length change in the bracket debonding group was 3.1 mm [17].

Scores of the adhesive remnant index (ARI)

The greater frequencies of residual adhesive material sensation at grades (0) and (1) by 75% were in the traditional bracket removal group. This suggests that debonding primarily occurs at the bracket/adhesive interface, which may be attributed to the application of debonding force at the bracket base and adhesive area, leading to pressure concentration on the bracket surface and bond failure at the bracket/adhesive level [4]. A greater frequency was observed for grades (3) and (4) within both the 2.5W diode group and the 5W diode group, indicating that debonding occurs primarily at the adhesive/bracket interface or within the adhesive. However, there was no statistically significant difference in the residual adhesive material distribution among the three study groups. These results are consistent with studies conducted by Knaup et al. and Heravi et al., which found no statistically significant differences in adhesive material distribution among different bracket removal techniques [15,17]. However, these results disagreed with the study by Sedky and Gutknecht [13], which showed that a significant portion of the adhesive is removed along with the bracket when using the Er, Cr: YSGG laser, thus reducing the adhesive material’s sensation value. This discrepancy may be attributed to the use of the Er, Cr: YSGG laser in the study by Sedky, while a diode laser was used in this study [13].

The pulp chamber temperature

The average temperature raised in the 2.5W diode group was c2.37° [M.Y.1], while it was c3.60°in the 5W diode group. This confirms the safety of the laser debonding method, which may be attributed to the chosen application time and other criteria.

The current study's findings align with those of the study conducted by Dostalova et al., who measured the temperature rise within the tooth during the application of an ER: YAG laser for debonding metal and ceramic brackets using a thermal camera. The temperature rise ranged from c2.0° to c3.2°, not exceeding the thermal threshold for pulp damage [25]. This is consistent with the study by Stein et al., who investigated the temperature rise within the pulp chamber and solid tissues experimentally before and after the application of the diode laser [26]. The maximum temperature rise within the pulp chamber was c2.37°, not exceeding the estimated safe thermal threshold of 5.5°C. They confirmed that debonding ceramic brackets with a diode laser does not pose a risk to the vitality of the dental pulp [26].

Limitations

Although this study is the first to compare the length, direction, number, and formation of cracks, the score of the ARI, and the pulp chamber temperature in the case of metal bracket debonding using a 5-watt and 2.5-watt diode laser, some limitations were encountered. The assessments were conducted using an X-20 magnification stereomicroscope instead of an electron microscope, enabling the examination of crack depth before and after debonding. Other types of brackets, such as ceramic or crystal brackets, were not evaluated, and the effect of bracket removal was only assessed on molars, not on other teeth, such as incisors or canines.

## Conclusions

Laser-assisted debonding can effectively reduce the risk of enamel damage during the debonding procedure. Importantly, the application of a diode laser did not impact the amount of adhesive residue left on the tooth surface following debonding. The increase in pulp chamber temperature was below the assumed temperature threshold (5.5° C) at which pulpal damage may occur. Therefore, laser debonding could facilitate the debonding of metal brackets at the end of orthodontic treatment.
